# Draft genome sequence of *Streptomyces* sp. Sce081 isolated from the nest of wasp *Sceliphron destillatorium* (Illiger, 1807)

**DOI:** 10.1128/mra.01125-24

**Published:** 2025-02-25

**Authors:** Sofiia Pytel-Huta, Anna Kachor, Andriy Zatushevsky, Markiyan Samborskyy, Yuriy Rebets

**Affiliations:** 1 Department of Zoology, Ivan Franko National University of Lviv 112865 , Lviv, Ukraine; 2 Explogen LLC, Lviv, Ukraine; University of Maryland School of Medicine, Baltimore, Maryland, USA

**Keywords:** *Streptomyces*, actinomycetes, genome, wasp

## Abstract

Actinobacteria are often involved in ecological interactions with insects. *Streptomyces* sp. Sce081 with notable antifungal activity was isolated from the nest of wasp *Sceliphron destillatorium* (Illiger, 1807). Here, we report the draft sequence and analysis of the *Streptomyces* sp. Sce081 genome.

## ANNOUNCEMENT

Insect-associated actinomycetes are a promising source of new bioactive compounds. For instance, sceliphrolactam (1), dentigerumycin (2), and selvamicin (3) isolated from such actinomycetes are proposed to play an important role in the host insects' biology. Strain *Streptomyces* sp. Sce081 was isolated from the nest material of mud-dauber wasp *Sceliphron destillatorium* (Illiger, 1807). These wasps lay their eggs into nests which are built from mud and clay and filled with paralyzed spiders. The conditions inside the nest are favorable for fungi; however, nests are typically clean of fungal contamination. This antifungal activity may result from bacteria associated with wasps or nest material.

**Fig 1:**
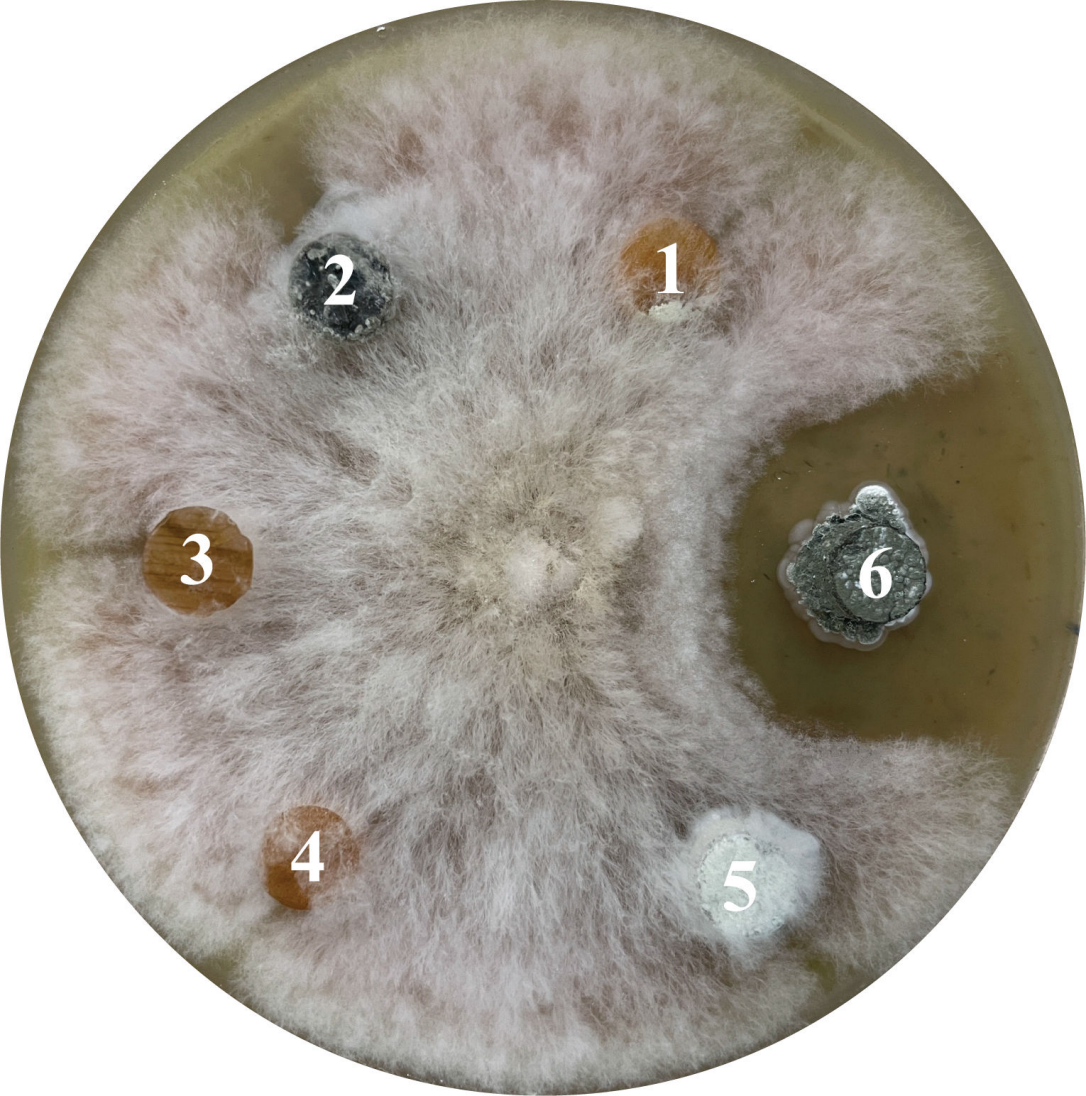
Plaque diffusion antifungal activity test of actinobacteria isolates: (1) – Sce080; (2) – Sce079; (3) – Sce078; (4) – Sce083; (5) – Sce082; (6) – Sce081. Strains were cultured on MS agar for 7 days at 30 ° C. Plaques were cut off and placed on fungal test culture. Plates incubated for 5 days at 30 ° C.

The nest was collected from the attic of the house in Velyki Hrybovychi, Lviv region, Ukraine (Global Positioning System coordinates: N 49°54'38.7" E 24°02'16.7"). The nest was identified by visual peculiarities to belong to *Sceliphron destillatorium* wasp. The Sce081 isolate was obtained by dilution plating (10^−2^) of an aqueous suspension of a ground nest on HVA medium (4) and incubated at 30°C for 30 days. The used approach was not selective for actinobacteria, and the isolate Sce081 was chosen based on its morphological properties. The isolate exhibited antifungal activity when tested by agar plaque diffusion assay (Fig. 1). For genomic DNA extraction, Sce081 was cultured on MS-agar medium (5) at 30°C for 72 hours. Genomic DNA was extracted using the NucleoSpin Microbial DNA kit (Macherey-Nagel, Germany) following the manufacturer’s instructions. The Sce081 isolate was identified by Sanger sequencing of the 16S rRNA gene using universal primers 27F and 1492R (6). The obtained sequence (PQ427446) alignment to the NCBI refseq rRNA database using BLAST v2.15.0 (7) resulted in the closest match (100% identity) to be *Streptomyces prasinus* NBRC 13479 (NR_041230). The library preparation for the Illumina Novaseq 6000 system was performed using a TruSeq DNA PCR-Free Kit and sequenced in a 2 × 150 bp paired-end configuration. The reads QC was performed using FastQC (8). The genome coverage was x319. The adapters were trimmed with fastq_miseq_trimmer v.t19, and the data were assembled with Newbler v3.0 (F. Hoffmann-La Roche AG, Switzerland) using default parameters. The assembly yielded 114 contigs, with a total length of 7,933,994 bp, an *N_50_* value of 127,902 bp, and an *L_50_* value of 21. The Sce081 genome has a G+C content of 72.22%. A total of 6,540 protein-coding genes, 68 tRNA and 2 rRNA genes, 3 non-coding RNAs, and 211 pseudogenes were predicted using the NCBI Prokaryotic Genome Annotation Pipeline v6.7 (9).

The genome analysis with antiSMASH v7.1.0 (10) predicted 34 regions encoding secondary metabolite biosynthetic gene clusters (BGC) with some being fragments of large clusters incorrectly assembled due to the repetitive nature of the corresponding sequences. The observed antifungal activity of the strain could be at least partially attributed to the presence of the polycyclic tetramine macrolactams BGC which is a very well-known antifungal metabolite efficiently produced in laboratory conditions (BGC0001043) (11). However, the majority of BGCs predicted within the genome of *Streptomyces* sp. Sce081 are coding for potentially new natural products, giving a promise for new biologically active compound discovery.

## Data Availability

The genome sequence of *Streptomyces* sp. Sce081 is available in NCBI GenBank under genome accession number JBHNYJ000000000, BioProject accession number PRJNA1164031, and BioSample accession number SAMN43882347. The raw sequence data are available under Sequence Read Archive accession numbers SRR30785061.
